# Phenotypic Diversity of Seminal Root Traits in Bread Wheat Germplasm from Different Origins

**DOI:** 10.3390/plants11212842

**Published:** 2022-10-25

**Authors:** Isabel P. Pais, Rita Moreira, José N. Semedo, Fernando H. Reboredo, Fernando C. Lidon, José Coutinho, Benvindo Maçãs, Paula Scotti-Campos

**Affiliations:** 1 Instituto Nacional de Investigação Agrária e Veterinária, I.P., Quinta do Marquês, Av. República, 2784-505 Oeiras, Portugal; 2 GeoBioTec Research Center, Faculdade de Ciências e Tecnologia, Campus da Caparica, Universidade Nova de Lisboa, 2829-516 Caparica, Portugal; 3 Earth Sciences Department, Faculdade de Ciências e Tecnologia, Campus da Caparica, Universidade Nova de Lisboa, 2829-516 Caparica, Portugal; 4 Instituto Nacional de Investigação Agrária e Veterinária, I.P., Estrada Gil Vaz, Ap. 6, 7350-901 Elvas, Portugal

**Keywords:** seminal roots, root growth angle, seminal root number, seminal root length

## Abstract

Bread wheat (*Triticum aestivum* L.) is a major staple crop, and more adapted varieties are needed to ensure productivity under unpredictable stress scenarios resulting from climate changes. In the development of new genotypes, root system traits are essential since roots have a key function in water and nutrient uptake, and root architecture determines the plant’s ability to spatially explore the soil resources. Genetic variation in wheat root system may be assessed at the early stages of development. This study evaluates in vitro and at the seedling stage, the genetic diversity of root growth angle (RGA), seminal root number (SRN), and radicle length (RadL) in 30 bread wheat genotypes from different origins and belonging to distinct evolutive or breeding groups. SRN and RadL were analyzed at 1, 2, 3 and 6 days after sowing (DAS) and RGA was measured through the angle between the first pair of seminal roots. A large variability was found in RGA values that ranged from 63° to 122°. Although differences were found between genotypes within the same groups, the narrower angles tended to occur among landraces, while the higher RGA values were observed in advanced lines and Australian varieties. Differences were also observed as regards the SRN (1.0–3.0, 2.7–4.7, 3.2–5.0 and 4.4–6.3 at 1, 2, 3 and 6 DAS, respectively) and RadL (0.1–1.5, 2.1–5.0, 4.0–7.5 and 5.1–13.7 cm at 1, 2, 3 and 6 DAS, respectively). Genetic variability in root traits at seedling stage allows more rapid selection of genotypes better adapted to environmental and soil constraints, necessary to Portuguese Wheat Breeding Program. It will also contribute to the definition of wheat ideotypes with improved performance under Mediterranean climate conditions.

## 1. Introduction

Bread wheat (*Triticum aestivum* L.) is one of the world’s staple crops, with high economic and social impact for human food and livestock feed. Under current climate change scenarios, it is a great challenge to breed genotypes that can cope with the upcoming environmental conditions, such as water deficit, and rising temperature and CO_2_ [1,2]. 

A decrease in food security is expected as a result of a rise in food demand combined with a decrease in arable land per capita (due to population growth, urbanization, and soil degradation) [3], shortages of global fertilizer stocks [4], as well as the unpredictable and uneven occurrences of extreme climatic events related to climate changes across the globe [5]. For the Mediterranean basin, losses of 60% in bread wheat yield have been estimated due to the expected effects of global warming [6,7]. On the Iberian Peninsula, which has been identified as a significant climate change hotspot, precipitation and temperature patterns have already impacted wheat production [8].

In the previous century, the green revolution was primarily driven by dwarf plant varieties selected for their response to soil fertility, fertilizer application, and water availability, allowing population growth to keep pace. Since the 1990s, lower yield increases have been achieved [4]. The next “agricultural revolution” must select plants with plasticity to maintain yields under less-than-optimal conditions. Furthermore, breeding new varieties must also take into account the effectiveness of nutrient acquisition by crops.

Roots play a crucial role in water and nutrients uptake by plants, in addition to metabolite storage, anchorage and mechanical support [9]. Under some abiotic stresses, such as drought, waterlogging, nutrient deficiency, high salinity, and/or mineral toxicity, the initial negative impact on a plant, as well as the first response, occurs at roots level [10] despite the effects that can be expressed later on above-ground plant organs [11,12]. Root system architecture (RSA) comprises the shape and spatial arrangement of a plant’s root system within the soil and sets the plant’s ability to spatially explore it [9]. Determined by the interaction of plant genetics and soil characteristics [9], RSA also differs considerably between wheat genotypes [13] and, consequently, in their pattern of water and nutrient uptake.

Water and nutrients availability have a profound effect on plant growth. These resources are heterogeneously distributed in the soil, with the greatest variations occurring with depth [14]. In the rhizosphere, the top layer is typically more nutrient-rich and holds fewer mobile elements such as phosphorus and potassium [3]. Usually, it also has a lower water content [7] and higher oxygen concentrations [15]. Some authors reported that in flooded soils, genotypes with a shallow root system produced higher yields than genotypes with a deep root system [13], reflecting some tolerance to this stress. Advantages in obtaining O_2_ [15] as well as phosphorus, potassium, and ammonium which are relatively immobile in the soil [9] may account for this enhanced performance. On the other hand, genotypes with a deeper root system (RS) present a better ability to cope with water deficiency as they are more able to absorb water and soluble nutrients from the soil, such as nitrogen, calcium, and magnesium, which tend to move to deeper soil layers [16]. Additionally, deeper RS may improve soil structure and its carbon steady-state, as well as water and nutrient retention, thereby contributing to more sustainable crop production [17]. Being an important agronomic trait in acclimation to several environmental constraints [18], it is advantageous that RSA play an important role in wheat breeding programs, allowing the development of new varieties with a suitable root ideotype for each specific environment [9].

The root system begins as a single root generated during embryogenesis and develops as the plant matures [19]. The wheat root system consists of seminal (i.e., embryonic) and nodal roots that remain functionally active throughout the plant’s life cycle. Seminal roots are originated from the seed embryo and the latter from stem nodes [19]. Due to their ability to develop earlier and deeper into the soil, seminal roots may be as significant or even more important than nodal roots for yield maintenance. Additionally, under insufficient soil moisture, plants achieve maturity primarily via their seminal roots, since nodal roots do not form or their development is restricted [20,21]. 

The primary seminal root (or radicle) develops first during germination and emerges within 1–2 days after imbibition (DAI). At the scuttelar node of the embryonic hypocotyls, two pairs of lateral seminal roots emerge to form the seminal roots system in wheat. The first pair emerges within 1 to 4 DAI, followed by a second pair (5–9 DAI). Occasionally, a sixth seminal root may appear in 5–10 DAI [22,23,24].

In wheat, genotypic diversity in root traits has been found [25]. Taking into consideration the difficulties of accessing mature root systems in soil, it is possible to select genotypes based on traits that are expressed in the early stages of development [26]. In fact, at the seedling stage, seminal root growth, specifically root growth angle (RGA), is closely linked to the architecture of mature plant root systems [23,25]. Apart from RGA, these genetically determined features include the number of seminal roots (NSR) and the primary root length (RadL) [7,10,19,22]. 

The RGA has been used as an early screening tool in cereal breeding programs since it can serve as a proxy for gravitropic root system tendency [27]. It determines root distribution and elongation direction, i.e., whether a plant has a shallow or deep root system [7]. Narrow seminal root angles have been associated with deeper root systems that reach lower soil levels, which can be advantageous in drought conditions. Conversely, wide angles were related with superficial root systems that promote lateral root growth, resulting in some benefits under wetter conditions and artificial irrigation [7,28]. Some authors observed a significant genetic variation in wheat’s RGA, which was related to the genetic background and geographical adaptation of varieties, being a valuable breeding resource for boosting crop yield [23,25,26].

Regarding seminal roots number (SRN), domesticated wheats have a higher number than their wild relatives [22,24] and it has been suggested that this variation plays a role in wheat’s adaptation to water stress. Genotypes with higher numbers of seminal roots presented a larger root surface and a denser and deeper root system, which promotes soil resource exploitation [29]. However, a reduction in the number of seminal roots increases hydraulic resistance and slows early water usage, and soil water will be used in more critical periods such as flowering and grain filling [22]. Some studies have suggested that endosperm reserves are a determinant of SRN [30,31]; however, other authors refer that this trait is regulated by embryo-expressed rather than non-endosperm-expressed factors [22].

The total length of roots in the soil impacts the absorption of water and nutrients and the overall performance of the crop [21]. When plants are grown in soils with insufficient water or nutrient content, extensive root systems are essential. Therefore, the uptake efficiency of the root apparatus as well as the identification of root features at the seedling stage may lead to an earlier selection of genotypes which cope better with a variety of adverse environments.

The main goals of this study were to evaluate in vitro, at the seedling stage, the extent of potential genetic diversity for the root growth angle, seminal root number and radicle length in a set of 30 genotypes of *Triticum aestivum* L. from different origins and belonging to distinct evolutive or breeding groups. We expect to optimize a fast and low-cost methodology to identify early root features eventually related to the root architecture of mature plants, and their improved performance under specific environmental conditions.

Variability in root traits at the seedling stage will allow more efficient selection of genotypes better adapted to environmental and soil constraints and contribute to breeding strategies for Portuguese Wheat Breeding Program and obtention of wheat ideotypes with improved performance under Mediterranean climate conditions.

## 2. Results

### 2.1. Root Growth Angle

**Figure 1:**
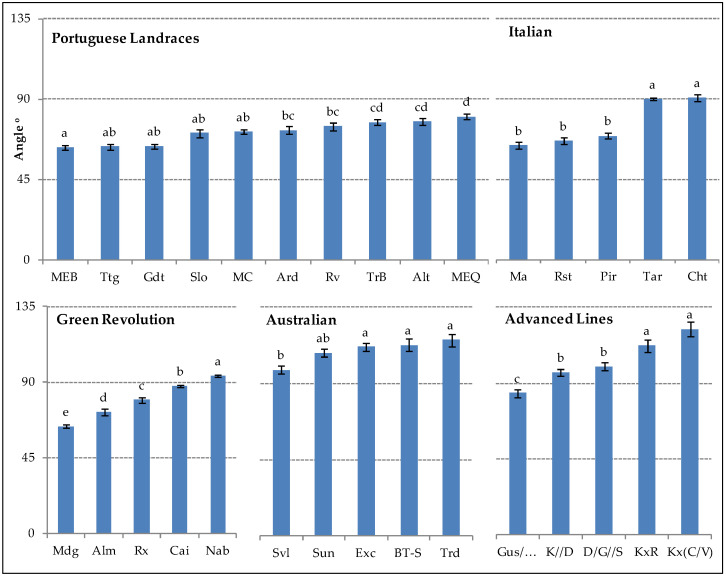
Root growth angle (RGA, °) between the first pair of lateral seminal roots of 30 bread wheat genotypes belonging to five evolutive/breeding groups. For each genotype, the mean values ± SE (*n* = 60) followed by different letters express significant differences (a, b, c, d, e) for a 95% confidence level. The highest value corresponds to the letter a.

Regarding Root Growth Angle (RGA) (Figure 1), phenotypic diversity was observed with values ranging from 63.1° (Portuguese Landrace, MEB) to 122.2° (Advanced Line, Kx(C/V). Variability was observed among groups. In the Portuguese Landraces, a tendency to narrow angles (63.1°–80.5°) was observed, whereas the Australians genotypes and Advanced Lines exhibited more open angles (98.0°–115.5° and 84.1°–122.2°, respectively). Despite the fact that there is variability within each of the studied groups, some exhibit greater variation between genotypes than others. The highest intra-group genotype difference was observed in the Advanced Lines (38.1°) followed by Green Revolution (30.1°), Italians (26.8°), Australians (17.5°) and Portuguese Landraces (17.4°).

Despite this smaller amplitude in Portuguese Landraces, significant differences in RGA values can be observed between genotypes, with MEB presenting the lowest value, followed by Ttg (63.6°), Gdt (63.8°), Slo (71.0°), MC (71.8°), Ard (72.8°), Rv (75.1°), TrB (77.2°), Alt (77.6°) and MEQ. In some genotypes with the introduction of Italian germplasm (Italian), Ma, Rst, and Pir RGA did not differ (63.7°, 66.3° and 69.3°, respectively) but nevertheless, they were distinct from Tar and Cht from the same group, which had greater angle values (89.7° and 90.5°, respectively). In the group with the introduction of CYMMYT germplasm (Green Revolution), all varieties had different RGA values, with Mdg having the narrowest angle (63.9°), followed by Alm (72.5°), Rx (79.6°), Cai (87.9°) and Nab (94.0°). In the Australian group, Svl showed a RGA value (98.0°) significantly different from Exc (111.4°), BT-S (112.5°) and Trd (115.5°) but similar to Sun (108.0°).

Diversity was also present within Advanced Lines group, with GUS/…showing the lowest RGA (84.1°), significantly differing from two other sub-groups: K/P//M/D and D/G//S (96.4 and 99.9°, respectively), and KxR and Kx(C/V) (112.3 and 122.2°, respectively).

**Figure 2:**
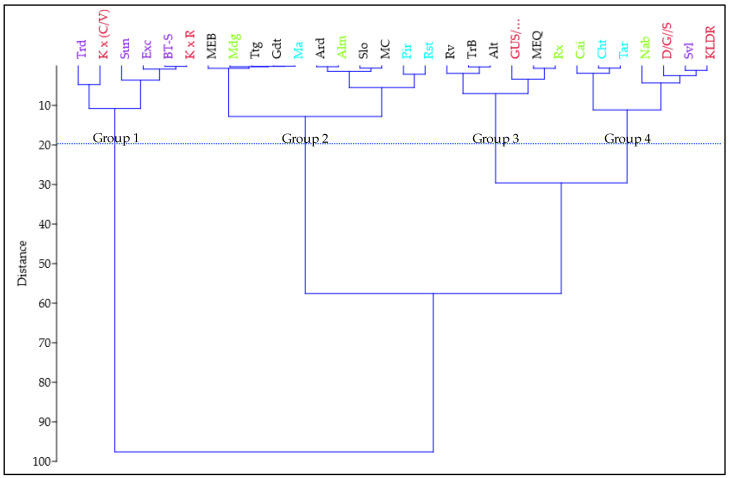
Dendrogram of hierarchical clustering using Ward’s method of 30 genotypes of *T. aestivum* L. based on the root growth angle at seedling stage. The horizontal line indicates the cut-off used to form the groups. Portuguese landraces (in black); Italians (in blue); Green Revolution (in green); Australians (in purple); Advanced Lines (in red).

Although RGA analysis was performed by group, it was evident that some genotypes had comparable values across groups. Using Ward’s method, a hierarchical cluster analysis was conducted in order to identify more homogenous groups of genotypes according to their similarity (Figure 2). 

RGA cluster analysis revealed that wheat genotypes were distributed in four distinct groups. Groups 1 (G1) and 3 (G3) included six genotypes each, whereas Group 2 (G2) and 4 (G4) consisted of eleven and seven genotypes, respectively. G1, with RGA values between 108.0 and 122.2°, was composed of four Australian genotypes and two advanced lines. G2 consisted of six Portuguese landraces, three Italian and two Green revolution genotypes, with RGA ranging from 63.1 to 72.8°. As regards G3, values from 75.1 to 84.1° were observed, corresponding to four Portuguese landraces, one Green revolution genotype and one Advanced Line. G4 comprised two Italian and two Green revolution genotypes, two Advanced Lines and one Australian variety, with RGA values from 87.9 to 99.9°.

### 2.2. Seminal Roots—Radicle Length

All genotypes exhibited a high germination rate (Table 1) throughout the study (40–100% at 1 DAS, 87–100% at 2 DAS and 97–100% at 3 and 6 DAS). Radicle length (RadL) showed some intra-group variation especially at the beginning of germination (1 DAS), as shown in Table 1. This was particularly evident among Portuguese Landraces, depicting values from 0.54 cm (Ard) and 1.49 cm (MEB). In the AL group values ranged from 0.13 cm (GUS/…, K/P//M/D and KxR) to 1.07 cm (D/G//S). These groups contained, respectively, the highest (MEB) and the lowest RadL1 values. However, after 6 days (RadL6) differences were attenuated and tended to similarity in all genotypes between the 5 groups regarding minimal (5.06–7.05 cm) and maximal RadL6 values (around 8.26–9.66 cm) except for the Slo genotype (PL group) which presented the highest value (13.85 cm).

### 2.3. Number of Seminal Roots

At the initial stage of germination (1 DAS), all genotypes had at least one seminal root (Table 2), and a maximum of 2–3 seminal roots. At this stage, the most homogeneous groups were Green Revolution and Australians, with values ranging from 2.63–3.0 and 2.90–3.0, respectively. However, this pattern is not evident at 6 DAS, when all groups exhibit comparable NSR (4.40–5.72 in PL; 4.73–5.47 in It; 4.93–5.47 in GR, 5.48–5.83 in Atrl and 5.10–5.80 in AL). 

Regarding the development of the sixth seminal root, all genotypes exhibited this trait except Gdt (Portuguese Landrace), although the incidence (%) differed. In the Australian and Advanced Lines groups, a frequency higher than 40% was observed in all genotypes except GUS/… (33.3%) with the highest values being observed in Trd (83.0%, Atrl) and KxR (80.9%, AL). On the last day of analysis (6 DAS), the Green Revolution group demonstrated the lowest proportion of sixth seminal root production, with values over 40% in only 2 of the 5 genotypes (Cai, 55.3% and Alm, 46.2%). These values were seen in 3 of 5 genotypes from the Italian group (Rst, 43.4%; Ma, 46.8% and Tar, 50.0%) and in 6 of the 10 Portuguese Landraces genotypes (Slo, 43.5%; Alt, 49.1%; Rv, 59.6%; Ard and MC, 71.9% and MEB, 76.7%).

### 2.4. Correlations between Parameters among Genotypes—Pearson Correlation Coefficient

As regards possible correlations of RGA and seed weight (SW) with several root traits most results showed coefficient values below 0.7, suggesting mainly the occurrence of weak or moderate relationship between these features (Table 3). Despite the variation RadL1 among genotypes, correlation between this trait and RGA did not show strong values. However, a strong positive correlation was found for RGA × RadL6 in two Green Revolution genotypes (Alm and Cai). This was also the case of RGA × NSR6, where positive coefficients were found for Alt (Portuguese Landrace), Cht (Italian) and Cai (Green Revolution). A negative correlation was observed for RGA × SW in Pir (Italian) and Rx (Green Revolution). A positive relationship between SWxRadL6 and SW × NSR6 was observed for Gdt (Portuguese Landrace). Negative SW × RadL6 and SW × NSR6 correlations were observed for Pir and Ma (Italians), respectively. The analysis also indicates a positive coefficient between RadL6 × NSR6 in Alm (Green Revolution) and Exc (Australian).

## 3. Discussion

The angle at which roots emerge from the seed and penetrate the soil determines root system architecture [27]. In several laboratory studies regarding RGA, results were consistent with field findings [32,33]. Work performed on seedlings showed RGA patterns were correlated with adult root morphology [34].

Results of RGA from the present study suggest that the majority of Portuguese Landraces depict narrower angles than genotypes from the other evolutive groups, in contrast with Australian and Advanced Lines resulting from recent breeding work, presenting wider RGA values. Narrow angles typically indicate a deeper root system, which enables plants to reach water and nutrients from lower soil layers and better water extraction capacity from the subsoil [27,35]. Portuguese Landraces are well adapted to the Mediterranean climate, which is characterized by a very long, hot and dry summer and concentrated precipitation in autumn and winter [36]. Lower RGA values would be beneficial to the yield stability under these extreme conditions. It is estimated that a 30 cm increase in root depth could capture an extra 10 mm of rainfall water during the critical grain filling stage [37] and that each additional mm of water extracted could generate an additional 55 kg ha^−1^ of grain yield [26]. 

Some authors suggest that Green Revolution strongly contributed to the reduction of wheat genetic diversity. Our results showed variability in RGA values within Green Revolution group, probably due to the presence of genotypes that result from cross-breeding of local germplasm with CIMMYT materials. According to Trethowan et al. [38], the open exchange of a large numbers of diverse materials across the wheat breeding programs around the world has resulted in genetic diversity that is at least as significant as that shown by CIMMYT-bred germplasm.

Wider angles of seminal roots are associated with superficial root systems where mass root is concentrated in the topsoil layers. This can play a crucial role in tolerance to wetter soil conditions, such as artificial irrigation or waterlogging, since this RSA facilitate water and nutrient uptake from a wider sub-surface area. Furthermore, root proliferation in superficial soil layers enhanced P capture and the access to oxygen, which are more available in this region of the rhizosphere [7,28,34,39]. 

The present results indicate that RGA variability exists between genotypes across groups, as well as within groups. This heterogeneity made cluster analysis challenging, with the obtention of four distinct groups showing some similarity between genotypes. Despite this, RGA cluster analysis confirmed that most Australian varieties and Advanced Lines genotypes depicted wider angles (G1 and G4), in contrast with Portuguese Landraces distributed in two other related groups (G2 and G3) together with some It and GR genotypes, presenting narrower RGA. 

The observed genetic diversity in wheat’s RGA appears to be partially related to the genetic background and local adaptation of varieties, which is in accordance with several authors [23,25,26]. However, niche similarity tests may be useful to elucidate whether geographical origin is linked to diversity, as performed in other species [40]. Most of the narrow angles were found in Portuguese Landraces genotypes (63.1–80.5°), which are part of a collection that represents the genetic diversity of regional wheat varieties from Portugal [36] but in the Italian and Green Revolution groups 3 of the 5 genotypes had angles between the same range. A strong negative correlation occurred between RGA and SW in only two genotypes; however, it may be due to the very early stage at which root traits were measured [34].

Regarding radicle length, results revealed some variability within groups. This was especially clear from the 1 DAS observations. The fast development of seminal roots may be beneficial in areas such as Mediterranean basin with limited upper layer soil moisture and where uneven rain distribution can cause young seedlings to become dehydrated [33]. In such environmental conditions, earlier root system deepening can increase water and nutrients uptake efficiency [21], and thus, contribute to the overall plant development. Since a deeper root is known to be a major component in improved drought avoidance [26], this characteristic, along with narrower RGA, could be considered suitable markers for drought resistance.

Between the genotypes under study, there were no differences in the maximum number of seminal roots (NSR). These results were consistent with the low variation in NSR in domesticated wheat which often reaches 5–6 as a maximum value, whereas it does not exceed 3 in wild wheat [22]. The higher number of seminal roots in domesticated wheat leads to a larger root surface area, a longer root system, and a higher root biomass, all of which improve the utilization of soil resources. In later stages of wheat growth, when water demand rises due to increased leaf area and higher transpiration, root area becomes a limiting factor, and an enlarged root system may be advantageous. However, fewer seminal roots can increase hydraulic resistance and slow down water usage making it available for a longer period [26]. In case of water limitation, adventitious roots development is inhibited [41], and in such case, seminal roots may sustain plant growth till maturity. Some studies have proposed that endosperm reserves are a key factor in determining NSR. Our findings do not support this statement since no correlation was found between SW and NSR in the great majority of the genotypes, suggesting that NSR is regulated by other mechanisms, as shown by Golan et al. [22].

Genetic variation in populations is needed to ensure appropriate breeding work. Root traits identification and characterization have advantages for progress to be made in root traits-based selection. This germplasm may be used for future breeding work allowing the selection of targeted root types. Knowledge concerning root angles and seminal roots in the different evolutive groups may also be used to design ideotypes more adapted to contrasting environments or watering conditions, namely drought, waterlogging, soil toxicity and nutrient availability.

## 4. Materials and Methods

### 4.1. Wheat Germplasm

Germplasm consisted in a set of 30 genotypes of *Triticum aestivum* L. from different origins and belonging to distinct evolutive or breeding groups: 

Ten Portuguese Landraces from an ancient wheat collection [42]: Alentejano (Alt); Ardito (Ard); Guaditano (Gdt); Mocho Cabeçudo (MC); Mocho de Espiga Quadrada (MEQ); Mocho de Espiga Branca (MEB); Tremês Branco (TrB); Transtagano (Ttg); Ruivo (Rv); Saloio (Slo).

Five varieties released in the period of 1950–1970 with the introduction of Italian germplasm, according to Almeida et al. [36]: Restauração (Rst); Chaimite (Cht); Mara (Ma); Pirana (Pir); Tarro (Tar).

Five varieties from the Post Green Revolution released between 1980–1989 with the introduction of CIMMYT germplasm, according to Almeida et al. [36]: (Cai); Nabão (Nab); Roxo (Rx); Mondego (Mdg); Almansor (Alm).

Five varieties from Australian germplasm: BT-Schomburgk (BT-S); Excalibur (Exc); Sunvale (Svl); Sunlin (Sun); Trident (Trd). 

Five Advanced lines from the Portuguese Cereal Breeding Program: Ducula/Gondo//Sokol (D/G//S); Katunga × (centauro/vega) (Kx(C/V); Kennedy x Roxo (KxR); KLDR/Pewit1//Milan/Ducula (K//D); GUS/3/Prl/Sara/Tsi/Vee#5/… (GUS/…).

Small amounts of certified seeds were supplied by the National Institute of Agrarian and Veterinary Research (INIAV, IP, Oeiras, Portugal), in the context of the National Breeding Program. Seeds multiplication was previously performed in growth chambers (Fitoclima 10,000 EHHF, ARALAB, Rio de Mouro, Portugal) under identical conditions of temperature (22/15 °C, day/night), irradiance (*ca.* 800 μmol m^−2^ s^−1^), relative humidity (70/75%, day/night), photoperiod (14 h) and CO_2_ (400 μL L^−1^) in 5 L pots with loamy clay soil harvested in the field, to ensure adequate seeds vigor and homogeneous germination capacity. The newly obtained seeds were used for in vitro evaluation assays.

### 4.2. Phenotypic Analysis

Seeds were previously sterilized with 96% ethanol for 10 s, rinsed with sterile water, soaked in 0.5% hypochlorite for 1 min, and then washed twice with water. Sowing was performed in polystyrene Petri dishes (12 × 12 cm) filled with sterilized agar (2%). Plates were positioned vertically to allow root growth in a vertical plane at 21 °C, in the dark (modified from [18]). Radicle length and seminal roots number were assessed at 1, 2, 3 and 6 DAS. RGA was evaluated by measuring the angle between the first pair of seminal roots with a minimum length of 3 cm. Phenotypic analysis was based on visual observations and manual measurements. RGA was assessed with a protractor and the seminal roots’ length with a ruler. For each genotype, 60 seeds were sown (6 per plate) with the germ end facing down. To evaluate the influence of seed size on the number of seminal roots, each grain was weighed before disinfection.

### 4.3. Statistical Analysis

Data were analyzed using a one-way ANOVA to evaluate the differences between genotypes within the evolutive/breeding group, followed by a Tukey’s test for mean comparisons. A 95% confidence level was adopted, which was performed always independently for each group. Hierarchical clustering analysis was performed using Ward´s method with PAST—PAlaeontological Statistics software., version 3, University of Oslo, Norway 

## 5. Conclusions

This study highlighted some variability in RGA at seedling stage, with narrower angles mainly occurring in Portuguese Landraces varieties, in contrast to larger angles found in Australians and Advanced Lines genotypes. Germplasm diversity identified in root traits may be used in breeding for yield stability/resilience under contrasting water situations occurring in Mediterranean regions namely drought (lower RGA) and waterlogging (higher RGA).

## Figures and Tables

**Table 1:** Radicle length (cm) and germination percentage (in parenthesis) at 1, 2, 3 and 6 days after sowing (DAS) of 30 bread wheat genotypes belonging to five evolutive/breeding groups. For each genotype, the mean values ± SE (*n* = 24–60) followed by different letters express significant differences (a, b, c, d, e, f) for a 95% confidence level. The highest value corresponds to the letter a. PL—Portuguese Landraces; It—Italians; GR—Green Revolution; Atrl—Australians; AL—Advanced Lines.

Group	Genotype	Radicle Length			
		1 DAS	2 DAS	3 DAS	6 DAS
PL	MEB	1.49 ± 0.02 ^a^ (80)	4.28 ± 0.10 ^cd^ (97)	5.42 ± 0.38 ^bc^ (97)	6.02 ± 0.49 ^ef^ (100)
	Ttg	1.14 ± 0.04 ^c^ (90)	4.52 ± 0.05 ^bc^ (97)	5.62 ± 0.10 ^bc^ (97)	6.31 ± 0.13 ^ef^ (100)
	Gdt	0.79 ± 0.06 ^d^ (70)	4.12 ± 0.09 ^de^ (90)	5.75 ± 0.12 ^bc^ (97)	9.49 ± 0.12 ^b^ (100)
	Slo	0.91 ± 0.01 ^d^ (100)	4.62 ± 0.10 ^ab^ (100)	7.65 ± 0.12 ^a^ (100)	13.85 ± 0.19 ^a^ (100)
	MC	1.29 ± 0.04 ^b^ (80)	4.68 ± 0.09 ^ab^ (87)	5.25 ± 0.09 ^cd^ (97)	5.24 ± 0.17 ^f^ (97)
	Ard	0.54 ± 0.04 ^f^ (90)	3.98 ± 0.03 ^de^ (100)	5.42 ± 0.09 ^bc^ (100)	6.59 ± 0.11 ^def^ (100)
	Rv	0.76 ± 0.02 ^de^ (97)	3.38 ± 0.02 ^g^ (100)	6.20 ± 0.15 ^b^ (100)	6.82 ± 0.40 ^cde^ (100)
	TrB	0.62 ± 0.02 ^e^ (97)	3.64 ± 0.11 ^fg^ (100)	5.64 ± 0.12 ^bc^ (100)	6.89 ± 0.14 ^cde^ (100)
	Alt	1.29 ± 0.12 ^b^ (97)	4.97 ± 0.06 ^a^ (100)	6.15 ± 0.14 ^b^ (100)	8.12 ± 0.41 ^bc^ (100)
	MEQ	0.90 ± 0.03 ^d^ (80)	3.78 ± 0.12 ^e^ (100)	4.55 ± 1.15 ^d^ (100)	7.92 ± 0.19 ^cd^ (100)
It	Ma	0.67 ± 0.02 ^ab^ (97)	5.01 ± 0.08 ^a^ (100)	6.15 ± 0.16 ^a^ (100)	8.85 ± 0.26 ^ab^ (100)
	Rst	0.53 ± 0.03 ^c^ (90)	4.45 ± 0.07 ^b^ (100)	5.45 ± 0.16 ^b^ (100)	6.86 ± 0.38 ^c^ (100)
	Pir	0.71 ± 0.04 ^a^ (100)	3.48 ± 0.07 ^c^ (100)	5.20 ± 0.10 ^b^ (100)	7.97 ± 0.45 ^bc^ (100)
	Tar	0.59 ± 0.03 ^bc^ (100)	4.62 ± 0.07 ^b^ (100)	5.99 ± 0.16 ^a^ (100)	9.63 ± 0.38 ^a^ (100)
	Cht	0.31 ± 0.02 ^d^ (47)	3.46 ± 0.10 ^c^ (100)	4.36 ± 0.12 ^c^ (100)	6.84 ± 0.43 ^c^ (100)
GR	Mdg	0.98 ± 0.04 ^a^ (100)	4.78 ± 0.06 ^a^ (100)	5.84 ± 0.15 ^a^ (100)	7.93 ± 0.23 ^b^ (100)
	Alm	0.46 ± 0.03 ^b^ (97)	2.44 ± 0.08 ^d^ (100)	3.96 ± 0.08 ^b^ (100)	7.55 ± 0.33 ^b^ (100)
	Rx	0.90 ± 0.03 ^a^ (97)	4.42 ± 0.06 ^b^ (97)	5.74 ± 0.19 ^a^ (97)	8.49 ± 0.34 ^ab^ (97)
	Cai	0.51 ± 0.03 ^b^ (90)	3.59 ± 0.06 ^c^ (97)	4.17 ± 0.14 ^b^ (97)	6.25 ± 0.24 ^c^ (97)
	Nab	0.49 ± 0.03 ^b^ (100)	4.20 ± 0.05 ^b^ (100)	5.26 ± 0.17 ^a^ (100)	9.8 ± 0.41 ^a^ (100)
Atrl	Svl	0.99 ± 0.02 ^a^ (98)	4.08 ± 0.06 ^ab^ (100)	5.02 ± 0.18 ^a^ (100)	7.47 ± 0.28 ^ab^ (100)
	Sun	0.51 ± 0.03 ^c^ (77)	2.80 ± 0.06 ^d^ (98)	4.84 ± 0.10 ^a^ (98)	5.06 ± 0.13 ^c^ (98)
	Exc	0.93 ± 0.04 ^b^ (100)	3.85 ± 0.08 ^bc^ (100)	4.84 ± 0.14 ^a^ (100)	7.42 ± 0.23 ^ab^ (100)
	BT-S	0.96 ± 0.02 ^a^ (100)	4.16 ± 0.06 ^a^ (100)	5.26 ± 0.19 ^a^ (100)	8.26 ± 0.30 ^a^ (100)
	Trd	0.82 ± 0.04 ^b^ (97)	3.68 ± 0.08 ^c^ (100)	4.90 ± 0.13 ^a^ (100)	7.08 ± 0.28 ^b^ (100)
AL	Gus/…	0.13 ± 0.01 ^c^ (80)	2.14 ± 0.11 ^b^ (98)	4.43 ± 0.06 ^b^ (100)	10.8 ± 0.43 ^a^ (100)
	K/P//M/D	0.13 ± 0.01 ^c^ (46)	2.28 ± 0.11 ^b^ (73)	4.68 ± 0.08 ^b^ (100)	7.05 ± 0.26 ^c^ (100)
	D/G//S	1.07 ± 0.01 ^a^ (100)	4.18 ± 0.06 ^a^ (100)	5.34 ± 0.20 ^a^ (100)	8.08 ± 0.30 ^abc^ (100)
	KxR	0.17 ± 0.01 ^bc^ (40)	2.35 ± 0.09 ^b^ (100)	4.67 ± 0.07 ^b^ (100)	9.02 ± 0.42 ^ab^ (100)
	Kx(C/V)	0.19 ± 0.01 ^b^ (80)	2.33 ± 0.07 ^b^ (97)	4.68 ± 0.08 ^b^ (98)	7.83 ± 0.35 ^bc^ (98)

**Table 2:** Number of Seminal Roots (NSR) and percentage of 6th seminal root formation (in parenthesis) at 1, 2, 3 and 6 days after sowing (DAS) of 30 bread wheat genotypes belonging to five evolutive/breeding groups. For each genotype, the mean values ± SE (*n* = 24–60) followed by different letters express significant differences (a, b, c, d, e, f) for a 95% confidence level. The highest value corresponds to the letter a. PL—Portuguese Landraces; It—Italians; GR—Green Revolution; Atrl—Australians; AL—Advanced Lines.

Group	Genotype	Number Seminal Roots			
		1 DAS	2 DAS	3 DAS	6 DAS
PL	MEB	2.92 ± 0.04 ^a^	4.22 ± 0.14 ^b^	4.52 ± 0.09 ^bc^	5.67 ± 0.09 ^ab^ (77)
	Ttg	2.93 ± 0.03 ^a^	3.03 ± 0.06 ^c^	3.87 ± 0.10 ^d^	5.20 ± 0.08 ^cd^ (28)
	Gdt	2.52 ± 0.11 ^b^	3.12 ± 0.04 ^c^	3.25 ± 0.07 ^e^	4.40 ± 0.09 ^f^ (0)
	Slo	3.00 ± 0.00 ^a^	4.73 ± 0.07 ^a^	4.72 ± 0.12 ^ab^ (19)	5.43 ± 0.06 ^abc^ (44)
	MC	2.92 ± 0.04 ^a^	4.65 ± 0.09 ^a^	4.83 ± 0.12 ^ab^ (26)	5.70 ± 0.06 ^ab^ (72)
	Ard	1.83 ± 0.13 ^c^	3.87 ± 0.10 ^b^	4.65 ± 0.09 ^abc^ (6)	5.72 ± 0.06 ^a^ (72)
	Rv	3.00 ± 0.00 ^a^	4.72 ± 0.10 ^a^	5.00 ± 0.05 ^a^ (6)	5.58 ± 0.07 ^ab^ (60)
	TrB	1.97 ± 0.12 ^c^	3.10 ± 0.04 ^c^	4.28 ± 0.09 ^c^	4.95 ± 0.06 ^de^ (9)
	Alt	2.65 ± 0.07 ^ab^	3.92 ± 0.09 ^b^	4.52 ± 0.10 ^bc^ (4)	5.35 ± 0.09 ^bc^ (49)
	MEQ	2.08 ± 0.12 ^c^	3.00 ± 0.07 ^c^	3.30 ± 0.08 ^e^	4.77 ± 0.09 ^e^ (6)
It	Ma	2.10 ± 0.11 ^b^	3.00 ± 0.00 ^c^	3.47 ± 0.10 ^b^	5.35 ± 0.09 ^a^ (47)
	Rst	1.33 ± 0.09 ^cd^	3.37 ± 0.09 ^b^	4.45 ± 0.09 ^a^ (4)	5.12 ± 0.11 ^ab^ (43)
	Pir	1.60 ± 0.10 ^c^	3.00 ± 0.00 ^c^	3.37 ± 0.08 ^b^ (3)	4.73 ± 0.10 ^b^ (10)
	Tar	2.67 ± 0.07 ^a^	3.95 ± 0.10 ^a^	4.55 ± 0.10 ^a^	5.47 ± 0.08 ^a^ (50)
	Cht	1.00 ± 0.00 ^d^	3.00 ± 0.00 ^c^	3.17 ± 0.05 ^b^	4.85 ± 0.12 ^b^ (29)
GR	Mdg	2.97 ± 0.02 ^a^	3.00 ± 0.00 ^b^	4.55 ± 0.12 ^a^ (7)	5.12 ± 0.08 ^ab^ (24)
	Alm	2.67 ± 0.07 ^b^	3.95 ± 0.11 ^a^	4.73 ± 0.07 ^a^	5.47 ± 0.06 ^a^ (46)
	Rx	2.97 ± 0.02 ^a^	3.00 ± 0.00 ^b^	3.22 ± 0.08 ^c^	4.93 ± 0.08 ^b^ (14)
	Cai	2.63 ± 0.09 ^b^	3.07 ± 0.05 ^b^	4.17 ± 0.10 ^b^	5.07 ± 0.14 ^b^ (55)
	Nab	3.00 ± 0.00 ^a^	3.00 ± 0.05 ^b^	3.45 ± 0.08 ^c^	4.98 ± 0.05 ^b^ (7)
Atrl	Svl	3.00 ± 0.00 ^a^	3.00 ± 0.00 ^b^	4.87 ± 0.04 ^a^	5.48 ± 0.07 ^b^ (48)
	Sun	2.93 ± 0.05 ^a^	3.07 ± 0.05 ^b^	4.55 ± 0.09 ^b^	5.60 ± 0.09 ^ab^ (70)
	Exc	3.00 ± 0.00 ^a^	3.13 ± 0.06 ^b^	4.85 ± 0.05 ^a^	5.63 ± 0.06 ^ab^ (63)
	BT-S	2.90 ± 0.05 ^a^	3.53 ± 0.10 ^a^	4.35 ± 0.10 ^b^	5.67 ± 0.07 ^ab^ (68)
	Trd	2.97 ± 0.02 ^a^	3.27 ± 0.12 ^ab^	4.97 ± 0.08 ^a^ (13)	5.83 ± 0.05 ^a^ (83)
AL	Gus/…	2.77 ± 0.08 ^a^	2.90 ± 0.05 ^b^	4.40 ± 0.11	5.30 ± 0.07 ^bc^ (33)
	K/P//M/D	2.07 ± 0.07 ^b^	2.65 ± 0.09 ^b^	4.00 ± 0.12 ^b^	5.70 ± 0.07 ^a^ (73)
	D/G//S	3.00 ± 0.00 ^a^	3.27 ± 0.09 ^a^	4.63 ± 0.08 ^a^	5.60 ± 0.06 ^ab^ (60)
	KxR	2.83 ± 0.07 ^a^	2.83 ± 0.07 ^b^	3.75 ± 0.09 ^b^	5.80 ± 0.06 ^a^ (81)
	Kx(C/V)	1.50 ± 0.11 ^c^	2.67 ± 0.08 ^b^	3.30 ± 0.10 ^c^	5.10 ± 0.11 ^c^ (40)

**Table 3:** Pearson correlation coefficient between the root growth angle with the radicle length 1 and 6 days after sowing (RGA × RadL1 and RGA × RadL6, respectively), with the seed weight (RGA × SW) and with the number of seminal roots 6 days after sowing (RGA × NSR6) as well as the correlation between seed weight with the radicle length at 6 DAS (SW × RadL6) and with number of seminal roots at 6 DAS (SW × NSR6). We also presented the value for Pearson correlation of radicle length at 6 DAS with the number of seminal roots at 6 DAS (RadL6 × NSR6); *n* = 58–60.

Group	Genotype	RGA × RadL1	RGA × RadL6	RGA × SW	RGA × NSR6	SW × RadL6	SW × NSR6	RadL6 × NSR6
PL	MEB	0.129	0.421	−0.111	0.176	−0.099	−0.030	0.698
	Ttg	0.186	0.121	−0.055	0.526	0.078	0.062	0.095
	Gdt	0.022	0.603	0.664	0.485	0.821	0.722	0.611
	Slo	0.296	0.389	0.096	0.405	−0.319	0.103	0.125
	MC	0.246	0.226	−0.385	0.467	−0.453	−0.482	0.633
	Ard	0.246	0.403	−0.342	0.157	0.073	0.133	0.400
	Rv	0.599	0.398	−0.089	0.672	0.104	−0.092	0.364
	TrB	0.276	0.667	−0.255	0.471	−0.003	−0.267	0.338
	Alt	0.461	0.535	−0.444	0.831	−0.438	−0.445	0.467
	MEQ	0.297	0.598	0.059	0.509	0.509	0.165	−0.032
It	Ma	0.336	0.531	−0.683	0.618	0.618	−0.713	−0.691
	Rst	−0.057	0.070	−0.155	0.435	0.185	−0.154	0.383
	Pir	0.180	0.356	−0.730	0.273	−0.703	−0.498	0.344
	Tar	0.483	0.167	−0.020	−0.205	−0.431	−0.585	0.468
	Cht	0.108	0.222	−0.108	0.735	0.050	−0.037	0.445
GR	Mdg	0.449	0.289	−0.428	0.389	−0.251	−0.297	0.204
	Alm	0.502	0.800	−0.242	0.679	−0.228	−0.153	0.730
	Rx	0.177	0.667	−0.780	0.605	−0.670	−0.611	0.434
	Cai	0.256	0.817	−0.374	0.762	−0.431	−0.459	0.685
	Nab	0.335	0.648	−0.059	0.474	−0.096	−0.189	0.586
Atr	Svl	0.115	0.091	−0.512	0.541	−0.394	−0.626	0.197
	Sun	0.223	0.246	0.145	0.189	−0.189	0.027	0.647
	Exc	0.502	0.534	−0.064	0.505	−0.031	0.047	0.710
	BT-S	0.359	0.399	−0.546	0.383	−0.607	−0.477	0.688
	Trd	0.404	0.449	0.002	0.246	−0.005	−0.096	0.486
AL	Gus/…	0.071	0.594	−0.371	0.694	−0.334	−0.239	0.499
	K/P//M/D	0.029	0.116	−0.284	0.370	−0.339	−0.293	0.594
	D/G//S	0.332	0.374	−0.525	0.485	−0.494	−0.387	0.688
	KxR	−0.067	0.089	−0.288	0.380	0.034	−0.362	0.010
	Kx(C/V)	0.203	0.598	−0.123	0.559	−0.212	−0.189	0.743

Positive correlations are displayed in blue and negative correlations in red. Different intensity of the color corresponds to different correlation coefficients. More intense or paler colors correspond to stronger or weaker correlations, respectively.

## Data Availability

Not applicable.

## References

[1] Martins M.Q., Rodrigues W.P., Fortunato A.S., Leitao A.E., Rodrigues A.P., Pais I.P., Martins L.D., Silva M.J., Reboredo F.H., Partelli F.L. (2016). Protective response mechanisms to heat stress in interaction with high [CO_2_] conditions in *Coffea* spp.. Front. Plant Sci..

[2] Semedo J.N., Rodrigues A.P., Lidon F.C., Pais I.P., Marques I., Gouveia D., Armengaud J., Semedo M.C., Martins S., Simoes M.C. (2021). Intrinsic non-stomatal resilience to drought of photosythetic apparatus in *Coffea* spp. can be strengthened by elevated air CO_2_. Tree Physiol..

[3] Lynch J.P. (2007). Roots of the second green revolution. Aust. J. Bot..

[4] St. Clair S.B., Lynch J.P. (2010). The opening of Pandora’s Box: Climate change impacts on soil fertility and crop nutrition in developing countries. Plant Soil.

[5] Pais I.P., Reboredo F.H., Ramalho J.C., Pessoa M.F., Lidon F.C., Silva M.M. (2020). Potential impacts of climate change on agriculture: A review. Emir. J. Food Agric..

[6] Sanchez-Garcia M., Álvaro F., Martín-Sánchez J.A., Sillero J.C., Escribano J., Royo C. (2012). Breeding effects on the genotype×environment interaction for yield of bread wheat grown in Spain during the 20th century. Field Crops Res..

[7] Uga Y., Kitomi Y., Ishikawa S., Yano M. (2015). Genetic improvement for root growth angle to enhance crop production. Breed. Sci..

[8] Bento V.A., Ribeiro A.F.S., Russo A., Gouveia C.M., Cardoso R.M., Soares P.M.M. (2021). The impact of climate change on wheat and barley yields in the Iberian Peninsula. Sci. Rep..

[9] Takahashi H., Pradal C. (2021). Root phenotyping: Important and minimum information required for root modeling in crop plants. Breed. Sci..

[10] Chen Y., Palta J., Prasad P.V.V., Siddique K.H.M. (2020). Phenotypic variability in bread wheat root systems at the early vegetative stage. BMC Plant Biol..

[11] Reboredo F., Henriques F. (1991). Some Observations on the Leaf Ultrastructure of *Halimione Portulacoides* (L.) Aellen Grown in a Medium Containing Copper. J. Plant Physiol..

[12] Reboredo F. (2001). Cadmium Uptake by *Halimione portucaloides*: An Ecophysiological Study. Bull. Environ. Contam. Toxicol..

[13] Oyanagi A., Kiribuchi-Otobe C., Yanagisawa T., Miura S., Kobayashi H., Muranaka S. (2004). Growth and grain yield of wheat experimental lines with deep and shallow root system in wet paddy fields. Jpn J. Crop Sci..

[14] Haque M.E., Oyanagi A., Kawaguchi K. (2012). Aerenchyma formation in the seminal roots of Japanese wheat cultivars in relation to growth under waterlogged conditions. Plant Prod. Sci..

[15] Omori F., Mano Y. (2007). QTL mapping of root angle in F_2_ populations from maize ‘B73’ × teosinte ‘*Zea luxurians*’. Plant Root.

[16] Gonçalves S.L., Lynch J.P. (2014). Raízes de plantas anuais: Tolerância a estresses ambientais, eficiência na absorção de nutrientes e métodos para seleção de genótipos. Doc. Embrapa.

[17] Kell D.B. (2011). Breeding crop plants with deep roots: Their role in sustainable carbon, nutrient and water sequestration. Ann. Bot..

[18] Nagel K.A., Lenz H., Kastenholz B., Gilmer F., Averesch A., Putz A., Heinz K., Fischbach A., Scharr H., Fiorani F. (2020). The platform GrowScreen-Agar enables identification of phenotypic diversity in root and shoot growth traits of agar grown plants. Plant Methods.

[19] Rich S.M., Watt M. (2013). Soil conditions and cereal root system architecture: Review and considerations for linking Darwin and Weaver. J. Exp. Bot..

[20] Maccaferri M., El-Feki W., Nazemi G., Salvi S., Canè M.A., Colalongo M.C., Stefanelli S., Tuberosa R. (2016). Prioritizing quantitative trait *loci* for root system architecture in tetraploid wheat. J. Exp. Bot..

[21] Sanguineti M.C., Li S., MacCaferri M., Corneti S., Rotondo F., Chiari T., Tuberosa R. (2007). Genetic dissection of seminal root architecture in elite durum wheat germplasm. Ann. Appl. Biol..

[22] Golan G., Hendel E., Espitia G.E.M., Schwartz N., Peleg Z. (2018). Activation of seminal root primordia during wheat domestication reveals underlying mechanisms of plant resilience. Plant Cell Environ..

[23] Hohn C.E., Bektas H. (2020). Genetic Mapping of Quantitative Trait Loci (QTLs) Associated with Seminal Root Angle and Number in Three Populations of Bread Wheat (*Triticum aestivum* L.) with Common Parents. Plant Mol. Biol. Rep..

[24] Pigolev A., Miroshnichenko D., Dolgov S., Savchenko T. (2021). Regulation of sixth seminal root formation by jasmonate in *Triticum aestivum* L.. Plants.

[25] Manschadi A.M., Hammer G.L., Christopher J.T., DeVoil P. (2008). Genotypic variation in seedling root architectural traits and implications for drought adaptation in wheat (*Triticum aestivum* L.). Plant Soil.

[26] Manschadi A.M., Christopher J., Devoil P., Hammer G.L. (2006). The role of root architectural traits in adaptation of wheat to water-limited environments. Funct. Plant Biol..

[27] Wasson A.P., Richards R.A., Chatrath R., Misra S.C., Prasad S.V.S., Rebetzke G.J., Kirkegaard J.A., Christopher J., Watt M. (2012). Traits and selection strategies to improve root systems and water uptake in water-limited wheat crops. J. Exp. Bot..

[28] Lynch J.P. (2013). Steep, cheap and deep: An ideotype to optimize water and N acquisition by maize root systems. Ann. Bot..

[29] Richard C.A.I., Hickey L.T., Fletcher S., Jennings R., Chenu K., Christopher J.T. (2015). High-throughput phenotyping of seminal root traits in wheat. Plant Methods.

[30] Bektas H., Waines J. (2020). Effect of Grain Size on The Root System Architecture of Bread Wheat (*Triticum aestivum* L.). Turk. J. Agric. Res.

[31] Robertson B.M., Waines J.G., Gill B.S. (1979). Genetic Variability for Seedling Root Numbers in Wild and Domesticated Wheats 1. Crop Sci..

[32] Alahmad S., El Hassouni K., Bassi F.M., Dinglasan E., Youssef C., Quarry G., Aksoy A., Mazzucotelli E., Juhász A., Able J.A. (2019). A major root architecture QTL responding to water limitation in durum wheat. Front. Plant Sci..

[33] Hendel E., Bacher H., Oksenberg A., Walia H., Schwartz N., Peleg Z. (2021). Deciphering the genetic basis of wheat seminal root anatomy uncovers ancestral axial conductance alleles. Plant Cell Environ..

[34] Rufo R., Salvi S., Royo C., Soriano J.M. (2020). Exploring the genetic architecture of root-related traits in Mediterranean bread wheat landraces by genome-wide association analysis. Agronomy.

[35] Leigh F.J., Wright T.I.C., Horsnell R.A., Dyer S., Bentley A.R. (2022). Progenitor species hold untapped diversity for potential climate-responsive traits for use in wheat breeding and crop improvement. Heredity.

[36] Almeida A., Maçãs B., Rodrigues V., Torrão M., Bonjean A.P., Angus W.J., Van Ginkel M. (2016). Wheat breeding: Country perspectives. The History of Wheat Breeding in Portugal. The World Wheat Book: A History of Wheat Breeding.

[37] Kirkegaard J.A., Lilley J.M. (2007). Root penetration rate—A benchmark to identify soil and plant limitations to rooting depth in wheat. Aust. J. Exp. Agric..

[38] Trethowan R.M., Reynolds M.P., Ortiz-Monasterio J.I., Ortiz R., Janik J. (2007). The Genetic Basis of the Green Revolution in Wheat Production. Plant Breeding Reviews.

[39] Lynch J.P. (2019). Root phenotypes for improved nutrient capture: An underexploited opportunity for global agriculture. New Phytol..

[40] Smýkal P., Hradilová I., Trnený O., Brus J., Rathore A., Bariotakis M., Das R.R., Bhattacharyya D., Richards C., Coyne C.J. (2017). Genomic diversity and macroecology of the crop wild relatives of domesticated pea. Sci. Rep..

[41] Steinemann S., Zeng Z., McKay A., Heuer S., Langridge P., Huang C.Y. (2015). Dynamic root responses to drought and rewatering in two wheat (*Triticum aestivum*) genotypes. Plant Soil.

[42] Vasconcellos J.C. (1933). Trigos Portugueses desde há Muito Cultivados no País.

